# Ocular surface disease signs and symptoms of glaucoma patients and their relation to glaucoma medication in Finland

**DOI:** 10.1177/11206721221144339

**Published:** 2022-12-13

**Authors:** Minna Parkkari, Petri Purola, Hannu Uusitalo

**Affiliations:** 1 Department of Ophthalmology, SILK, 7840 University of Tampere, Tampere, Finland; 2 Finnish Register of Visual Impairment, Finnish Federation of the Visually Impaired, Helsinki, Finland; 3 Tauh Eye Center, Tampere, Finland

**Keywords:** Ocular surface disease, glaucoma medication, preservative, dry eye sensation, active compounds, multicenter study

## Abstract

**Purpose:**

To evaluate the prevalence of ocular surface disease (OSD) signs and symptoms of glaucoma patients in private clinics in relation to topical glaucoma treatment and to compare them to non-glaucomatous population.

**Methods:**

A multicenter, cross-sectional study consisting of private ophthalmology clinic visits in southern Finland. Glaucoma patients had a diagnosis of primary open-angle glaucoma, pseudoexfoliation glaucoma, pigmentary glaucoma, or treated ocular hypertension. Control patients had no prior or current use of glaucoma medication. Recorded parameters included OSD signs and symptoms, used glaucoma medications during the past 6 months, and the product name, type, and duration of used antiglaucoma drugs.

**Results:**

Glaucoma patients (*n* = 564) showed higher severity of OSD sign parameters excluding Schirmer's test, as well as increased dry eye sensation compared to controls (*n* = 51). Beta-blockers and preservative-free prostaglandins had the smallest effect on all parameters. The increasing number of active compounds and administered eye drops per day showed an association with increasing severity of OSD signs, as well as dry eye and foreign body sensation. Duration of glaucoma medication showed no significant association with OSD signs or symptoms.

**Conclusion:**

Glaucoma patients show higher prevalence of OSD signs and dry eye sensation compared to non-glaucomatous population. The use of preserved glaucoma medication, as well as high number of active compounds and eye drops increase the severity of these parameters. There are significant differences between the types of glaucoma medication used, and therefore the selection of them is important especially in patients suffering from OSD.

## Introduction

Glaucoma is the worldwide leading cause of irreversible visual impairment.^1^ Globally, the number of people suffering from primary open-angle glaucoma (POAG) was expected to rise to 65.5 million by 2020.^2^ In Finland, the number of glaucoma patients is over 90 000, and it is expected to increase to 120 000 by 2040 because of the ageing of the population.^3–4^

Glaucoma is associated with ocular surface disease (OSD). They both are known co-morbidities in ageing populations, and several studies have investigated the prevalence and impact of this clinical issue.^5–10^ These studies demonstrate that even 74% of glaucoma patients suffer from the symptoms of dry eye, which is the most common form of OSD.^11^ The presence of OSD can affect the quality of life of glaucoma patients.^12–13^ It is further known that poor compliance to treatment can contribute to the progression of glaucoma and OSD.^14^

Benzalkonium chloride (BAC) is the most used preservative in eye drops. BAC is known to exert toxic properties in the conjunctival and corneal epithelia.^15–16^ Typical side effects of BAC include conjunctival hyperemia, lid redness, conjunctival and corneal fluorescein staining, as well as subjective sensations such as stinging, watering, photophobia, and burning.^13^ The outcome of glaucoma surgery may also be affected negatively by the previous chronic use of preserved antiglaucoma eye drops.^17–19^ Based on these findings, BAC can significantly contribute to ocular surface problems and the overall success of treatment in glaucoma patients.

To our knowledge, there is little information on the prevalence and severity of ocular signs and symptoms in outpatient patients in private practices, even though a significant proportion of glaucoma patients are treated by private ophthalmologists in Finland and other countries. Therefore, we investigated in this cross-sectional multicenter study the occurrence of symptoms and signs of OSD in a glaucoma patient cohort and their relation to glaucoma medication using standardized methods. For comparison, we also included a control group not suffering from glaucoma.

## Methods

### Patients

This study was based on a multicenter, cross-sectional, single visit study performed in 2011. The data were obtained from patients attending private ophthalmologist clinics, enrolled in consecutive order. The study and all used methods were performed in accordance with the tenets of the Declaration of Helsinki, and the protocol was approved by the Independent Ethics Committee of Tampere University Hospital and the Finnish Medicines Agency (ETL R11015M, date 9.2.2011). Written informed consent was obtained from all subjects prior to inclusion.

The data were collected by 13 investigators in 27 centers in southern Finland. The amount of control patients to be enrolled was one among the first 10 glaucoma patients and after that always two control patients among the next 20 glaucoma patients. Glaucoma patients eligible for the study were aged 18 years or more and had a diagnosis of POAG, pseudoexfoliation glaucoma (ExG), pigmentary glaucoma (PG), or treated OH. The eligible control patients were 40 years or older, were not suffering from glaucoma, and had no prior or current use of glaucoma medication. Subjects were excluded if they had had eye surgery performed within the last 6 months, had known allergy, alcoholism, drug abuse, or any other condition that could have influenced the studied parameters.

Patient demography (i.e., gender and age), relevant systemic diseases, and other pertinent information were assessed. Detailed information about these parameters is shown in Supplemental Table 1 and has been published previously.^20^ Ocular diagnoses were recorded separately for both eyes as none, OH, POAG, ExG, or PG. If the patient had POAG, ExG, or PG in one eye and OH or no diagnosis in the other, the patient was classified as a corresponding glaucoma patient. If the patient had ExG in the first eye and POAG in the other eye, the patient was determined as an ExG patient. Glaucoma medication(s) used during the past 6 months were recorded, including the product name, type, and duration of used antiglaucoma drug(s). The same variables were assessed in control patients except for the use of glaucoma medication.

### Ocular signs

We assessed six different ocular signs: eyelid redness, conjunctival redness/hyperemia, corneal and conjunctival fluorescein staining, fluorescein tear break-up time (fBut), and tear secretion. Presence of eyelid redness was evaluated and severity graded as 0 (none), 1 (mild), 2 (moderate), or 3 (severe). Conjunctival redness/hyperemia was assessed by using reference photos (SILK Conjunctival Redness Grading) and a five-point scale: 0 = none, 1 = mild, 2 = moderate, 3 = severe, 4 = very severe. fBut was measured by adding fluorescein dye to the eyes and the tear film was observed under the slit lamb. The time it took to form micelles i.e., dry spots to develop was recorded as the fBUT (in seconds). After fBut measurement, corneal and conjunctival fluorescein stainings were scored by using reference pictures (Oxford Grading scale). Corneal (score 0 to V) and conjunctival staining (score 0 to X) were evaluated separately. Tear secretion was measured with Schirmer I test (without anesthesia) by placing filter paper under the lower eyelid for five minutes. The amount of moisture was read from the test strip (in millimeters). Both eyes were tested at the same time.

The assessment of ocular signs was made individually for the right and left eyes of glaucoma and control patients. The worst eye values were recorded for further analyses. An overall signs score was calculated by summarizing all six ocular sign scores, with higher score indicating more severe overall status. If the patient had different duration of glaucoma medication for each eye, the duration of the worse eye was used.

### Ocular symptoms

Ocular symptoms were evaluated via a post-visit telephone call made by a single, independent study nurse. All questions were asked with a fixed protocol. Evaluated ocular symptoms included irritation/burning/stinging, itching, foreign body sensation, tearing, and dry eye sensation. Each symptom was scored as none (O), very mild (1), mild (2), moderate (3) or severe (4). Additionally, a summary score from all five symptoms was calculated, with higher score indicating more severe overall status.

### Statistical analyses

Because the distribution of the data was skewed, non-parametric Mann­­–Whitney *U* test was used for continuous variables. Spearman's rank correlation coefficient was used to assess the correlation between two continuous variables. A two-tailed *p* value of < 0.05 was selected to determine statistical significance. All statistical analyses were performed using R software version 3.5.1 (R Core Team, Foundation for Statistical Computing, Vienna, Austria).

## Results

A total of 568 glaucoma patients and 51 controls were enrolled across 27 study centers. Four glaucoma patients were excluded from the data analysis because of incomplete or missing medication information or diagnosis. Therefore, background information and ocular signs data were evaluated from 564 glaucoma patients. Data on ocular symptoms were obtained from 562 glaucoma patients. Background information and ocular data were evaluated from all 51 controls. The mean ages and gender distribution of the study population and glaucoma types are shown in Table 1.

**Table 1.** Ocular diagnosis, mean age, and gender of glaucoma patients and controls.

		*n* (%)	Mean age	% women
Glaucoma patients	All	564	70	68
	Primary open-angle glaucoma	451 (80)	69	67
	Pseudoexfoliation glaucoma	90 (16)	76	74
	Pigmentary glaucoma	12 (2)	61	67
	Ocular hypertension	11 (2)	66	64
Controls		51	64	55

When compared to controls, glaucoma patients showed statistically significant worsening in all ocular sign parameters and overall signs score (*p* < 0.001, Mann­­–Whitney *U* test) except in Schirmer's test (Table 2). When ocular symptoms were evaluated, glaucoma patients showed statistically significant increase in dry eye sensation (*p* = 0.040) and symptom sum (*p* = 0.024) compared to controls (Table 3). After age-adjustment, corneal fluorescein staining and fBUT showed no longer significant difference. On the contrary, itching appeared to show significant difference.

**Table 2.** Ocular signs in glaucoma patients and controls.

Ocular sign	Glaucoma patients *n* = 564; (SD)	Controls *n* = 51; (SD)	Difference GIaucoma–Control: Mann–Whitney, *P*	Age-adjusted difference: Mann–Whitney, *P*
Eyelid redness	0.9 (0.8)	0.04 (0.2)	**< 0.001**	**< 0.001**
Conjunctival redness (SILK scale)	1.9 (0.8)	1.2 (0.8)	**< 0.001**	**0.006**
Corneal fluorescein staining (Oxford scale)	1.3 (1.3)	0.8 (1.0)	**0.006**	0.66
Conjunctival fluorescein staining (Oxford scale, combined nasal & temporal)	2.8 (2.1)	2.0 (2.1)	**0.005**	**< 0.001**
fBUT (seconds)	5.2^a^ (4.2)	7.3^b^ (8.4)	**0.008**	0.31
Schirmer's test (millimeters)	10.8 (8.1)	11.9 (8.1)	0.36	0.55
Overall signs score	10.9 (4.7)	7.4 (4.8)	**< 0.001**	**0.015**

^a^ n = 557 ^b^n = 48.

SD: standard deviation.

**Table 3.** Ocular symptoms in glaucoma patients and controls.

Ocular symptom	Glaucoma patients *n* = 562	Controls *n* = 51	Difference GIaucoma–Control: Mann–Whitney, *P*	Age-adjusted difference: Mann–Whitney, *P*
Irritation/burning/stinging	0.69	0.57	0.41	0.22
Itching	0.62	0.39	0.18	**0.043**
Foreign body sensation	0.64	0.47	0.22	0.37
Tearing	0.37	0.35	0.85	0.20
Dry eye sensation	0.94	0.59	**0.040**	**0.023**
Symptom sum	3.26	2.37	**0.024**	**0.004**

Data on the ocular signs in different glaucoma medication types are shown in Table 4. There was statistically significant worsening between different medication types in most of the observed signs when compared to controls. However, no significant difference was found in Schirmer`s values. Beta-blockers and preservative-free prostaglandins had the least effect on these parameters, and they showed no significant effect on the overall sign score when compared to controls. Medication with three active compounds (beta-blocker and prostaglandin fixed-dose combination plus carbonic anhydrase inhibitor) and combinations with brimonidine showed the most severe impact on these parameters when compared to controls, as well as to beta-blockers (Supplemental Table 2a). Ocular symptoms in different glaucoma medication types are listed in Table 5. Preserved prostaglandin, medication with three active compounds (fixed combination with beta-blocker and other (than prostaglandin) plus prostaglandin), and combinations with brimonidine showed statistically significant increase in symptom sum. In addition, combination of beta-blocker and prostaglandin, medication with three active compounds (fixed combination with beta-blocker and other (than prostaglandin) plus prostaglandin), and combinations with brimonidine showed statistically significant increase in dry eye sensation. Only combinations with brimonidine showed statistically significant increase in symptom sum when compared to beta-blockers (Supplemental Table 2b). Glaucoma medication duration showed no significant association with OSD signs or symptoms.

**Table 4.** Ocular signs related to glaucoma medication.

Medication	Number of patients (%)	Eyelid redness	Conjunctival redness (SILK scale)	Corneal fluorescein staining (Oxford scale)	Conjunctival fluorescein staining (Oxford scale, combined nasal & temporal)	fBUT (seconds)^a^	Schirmer's test (millimeters)	Overall signs score
Prostaglandin, all	204 (36)	**0.8**	**1.8**	0.9	2.5	6.3	12.4	9.9 **
Preserved prostaglandin	160 (28)	**0.8**	**1.9**	1.0	2.4	5.9	12.1	**10.1**
Preservative-free prostaglandin	44 (8)	**0.8**	1.6 *	0.8	2.8 *	7.5	13.4	9.4
Beta-blocker and prostaglandin fixed-dose combination	154 (27)	**1.0**	**2.0**	**1.5**	2.6	4.9 *	12.5	**11.2**
Beta-blocker and other (than prostaglandin) fixed-dose combination plus prostaglandin	58 (10)	**1.3**	**2.1**	1.2 *	3.4 **	5.3	12.1	**12.0**
Beta-blocker and prostaglandin fixed-dose combination plus carbonic anhydrase inhibitor	37 (7)	**1.1**	**2.0**	**2.3**	**3.4**	3.2 **	14.0	**13.4**
Beta-blocker	34 (6)	0.2 *	1.4	1.1	2.7	6.6	12.0	8.8
Beta-blocker and other (than prostaglandin) fixed-dose combination	25 (4)	0.4 **	**1.6**	1.4 *	3.3 **	6.5	10.4	10.4 **
Prostaglandin plus carbonic anhydrase inhibitor	16 (3)	**1.2**	**2.2**	1.6 *	2.5	5.4	14.7	11.7 *
Beta-blocker and prostaglandin	14 (4)	**0.7**	1.6 *	**0.6**	2.6	8.3	9.0	9.3
Combinations with brimonidine	22 (3)	**1.4**	**2.8**	**2.3**	3.7 **	2.9 *	11.9	**15.1**
Total	564							
Controls	51	0.04	1.2	0.8	2.0	7.3^b^	11.9	7.4

^a^ *n* = 557.

^b^ *n* = 48.

*Denotes statistical significance (Mann–Whitney) compared to controls with *p* < 0.05.

**Denotes statistical significance compared to controls with *p* < 0.01.

Bolded denotes statistical significance compared to controls with *p* < 0.001.

**Table 5.** Ocular symptoms related glaucoma medication.

Medication	Number of patients (%)	Irritation/burning/stinging	Itching	Foreign body sensation	Tearing	Dry eye sensation	Symptom sum
Prostaglandin, all	202 (36)	0.64	0.67	0.60	0.27	0.88	3.05
Preserved prostaglandin	158 (28)	0.65	0.71	0.68	0.30	0.89	3.23 *
Preservative-free prostaglandin	44 (8)	0.59	0.52	0.32	0.14	0.86	2.43
Beta-blocker and prostaglandin fixed-dose combination	154 (27)	0.68	0.69	0.68	0.28	0.90	3.23
Beta-blocker and other (than prostaglandin) fixed-dose combination plus prostaglandin	58 (10)	0.76	0.48	0.62	0.43	1.12 *	3.41 *
Beta-blocker and prostaglandin fixed-dose combination plus carbonic anhydrase inhibitor	37 (7)	0.84	0.78	0.70	0.62	0.78	3.73
Beta-blocker	34 (6)	0.59	0.26	0.59	0.59	0.85	2.85
Beta-blocker and other (than prostaglandin) fixed-dose combination	25 (4)	0.44	0.44	0.28	0.72	0.80	2.68
Prostaglandin plus carbonic anhydrase inhibitor	16 (3)	1.20	0.53	0.40	0.40	1.00	3.53
Beta-blocker and prostaglandin	14 (4)	0.43	0.50	0.43	0.36	1.36 *	3.07
Combinations with brimonidine	22 (3)	0.96	0.73	1.46 **	0.64	**1.55**	5.32 **
Total	562						
Controls	51	0.57	0.39	0.47	0.35	0.59	2.37

*Denotes statistical significance (Mann–Whitney) compared to controls with *p* < 0.05.

**Denotes statistical significance compared to controls with *p* < 0.01.

Bolded denotes statistical significance compared to controls with *p* < 0.001.

**Figure 1.**
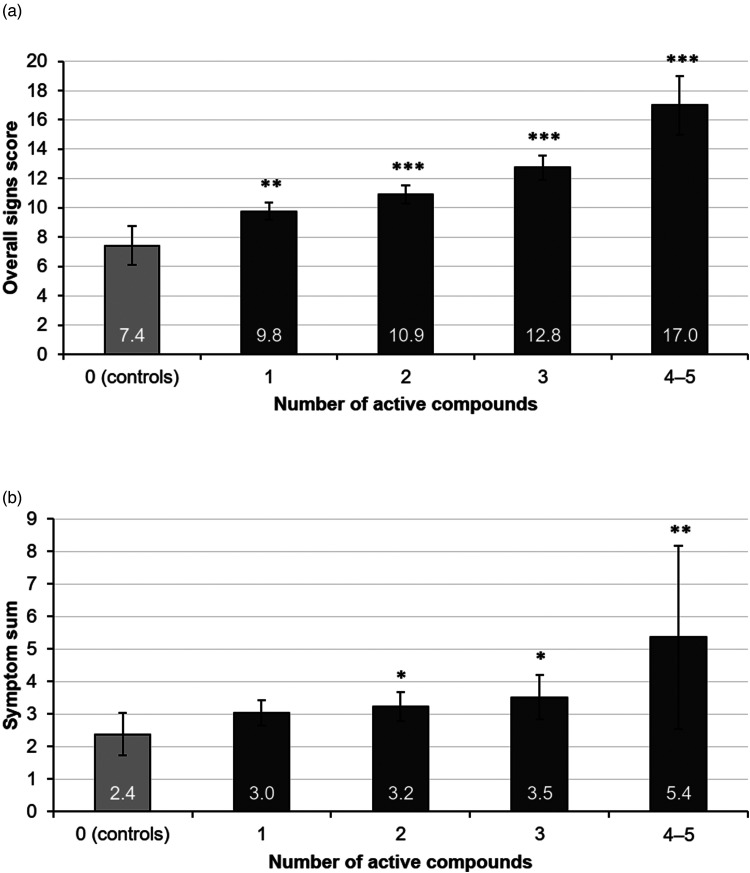
Mean of overall signs score (a) and symptom sum (b) with 95% confidence intervals related to number of active compounds in glaucoma patients compared to controls. *Denotes statistical significance (Mann–Whitney) compared to controls with *p* < 0.05. **Denotes statistical significance compared to controls with *p* < 0.01. ***Denotes statistical significance compared to controls with *p* < 0.001.

The relation of the number of used active compounds in glaucoma medication and ocular signs and symptoms are shown in Tables 6 and 7. One active compound showed least impact on the parameters when compared to controls, as well as to groups with more active compounds (Supplemental Table 3 and Supplemental Figure 1). For most of the glaucoma patients (80%), the glaucoma treatment consisted of one or two active compounds. As the number of active compounds increased, all subtypes of ocular signs worsened significantly except Schirmer`s test. For symptoms, foreign body sensation, dry eye sensation, and symptom sum increased significantly along with the increased number of active compounds. In Figure 1(a) and 1(b) are shown the overall signs score and the symptom sum with 95% confidence intervals according to the number of active compounds. There was a weak correlation (*r* = 0.27, *p* < 0.001) between the increasing number of active compounds and the overall signs score.

**Table 6.** Ocular signs related to number of active compounds in glaucoma medication.

Number of active compounds	Number of patients (%)	Eyelid redness	Conjunctival redness (SILK scale)	Corneal fluorescein staining (Oxford scale)	Conjunctival fluorescein staining (Oxford scale, combined nasal & temporal)	fBUT (seconds)^a^	Schirmer's test (millimeters)	Overall signs score
1	241 (43)	**0.7**	**1.8**	0.95	2.5	6.3	12.3	9.8 **
2	207 (37)	**0.9**	**1.9**	1.3 **	2.7 *	5.3	12.2	**10.9**
3	102 (18)	**1.2**	**2.1**	**1.7**	**3.4**	4.3 **	12.6	**12.8**
4–5	14 (2)	**1.6**	**3.1**	**2.8**	**4.6**	2.6 **	12.6	**17.0**
Total	564							
0 (controls)	51	0.04	1.2	0.8	2.0	7.3^b^	11.9	7.4

^a^ *n* = 557.

^b^ *n* = 48.

*Denotes statistical significance (Mann–Whitney) compared to controls with *p* < 0.05.

**Denotes statistical significance compared to controls with *p* < 0.01.

Bolded denotes statistical significance compared to controls with *p* < 0.001.

**Table 7.** Ocular symptoms related to number of active compounds in glaucoma medication.

Number of active compounds	Number of patients (%)	Irritation/burning/stinging	Itching	Foreign body sensation	Tearing	Dry eye sensation	Symptom sum
1	239 (43)	0.63	0.61	0.60	0.32	0.88	3.04
2	207 (37)	0.69	0.65	0.60	0.36	0.94	3.23 *
3	102 (18)	0.75	0.59	0.67	0.52	1.00 *	3.52 *
4–5	14 (2)	1.14	0.57	1.64 **	0.36	1.64 **	5.36 **
Total	562						
0 (controls)	51	0.57	0.39	0.47	0.35	0.59	2.37

*Denotes statistical significance (Mann–Whitney) compared to controls with *p* < 0.05.

**Denotes statistical significance compared to controls with *p* < 0.01.

**Figure 2.**
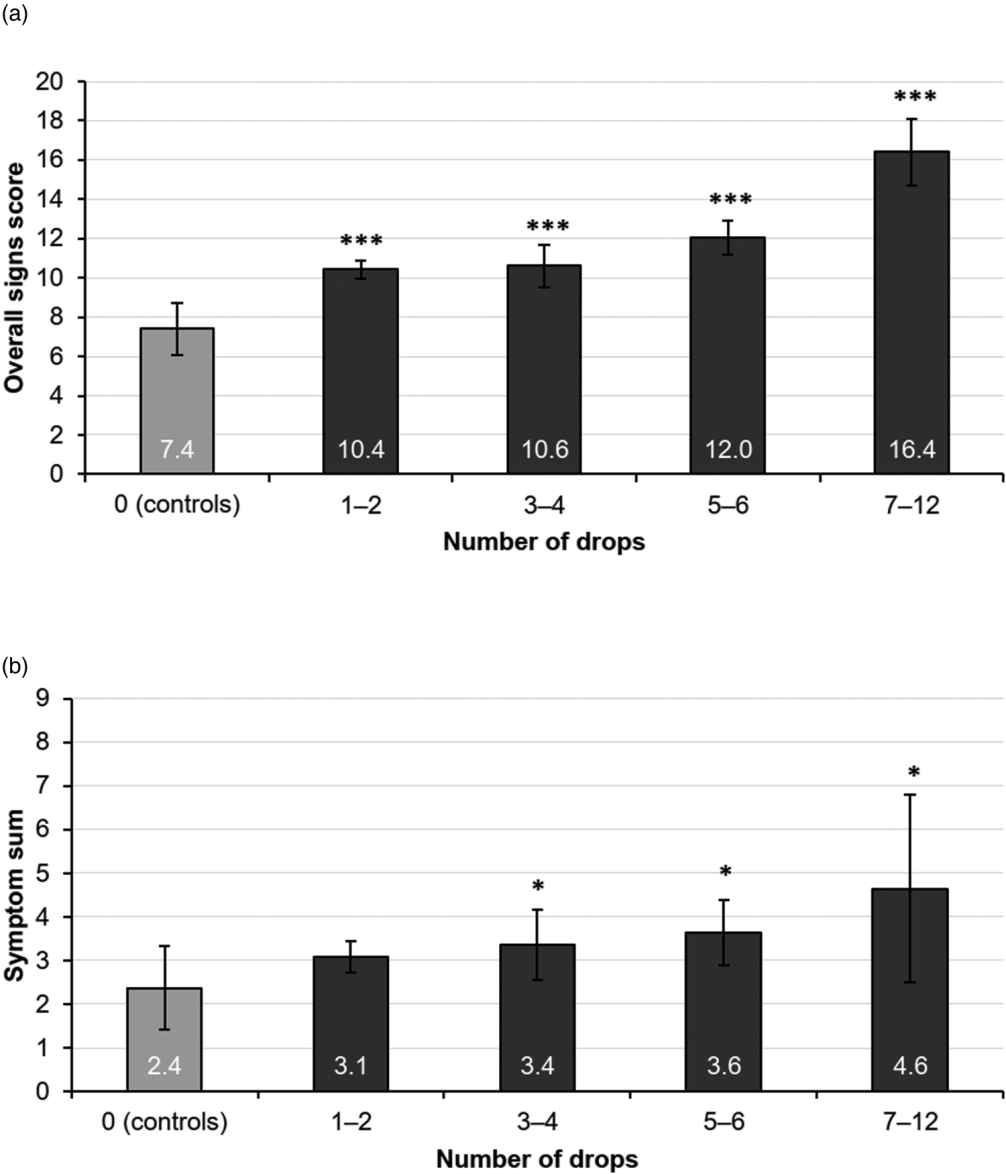
Mean of overall signs score (a) and symptom sum (b) with 95% confidence intervals related to number of administered eye drops per day among glaucoma patients compared to controls. *Denotes statistical significance (Mann–Whitney) compared to controls with *p* < 0.05. **Denotes statistical significance compared to controls with *p* < 0.01. ***Denotes statistical significance compared to controls with *p* < 0.001.

Tables 8 and 9 represent the ocular signs and symptoms related to the number of drops administered daily among the glaucoma patients. Group with 1–2 drops per day showed least impact on the parameters when compared to controls, as well as to groups with more drops (Supplemental Table 4 and Supplemental Figure 2). Most of the glaucoma patients (67%) instilled 1–2 glaucoma drops per day. All observed signs values worsened significantly along with the increasing number of drops except the values of Schirmer`s test. Foreign body sensation, dry eye sensation, and symptom sum increased significantly alongside with the number of drops. The relation of the number of drops and the overall signs score and the symptom sum is shown in Figures 2(a) and (b). There was a weak correlation (*r* = 0.15, *p* < 0.001) between the increasing number of drops and the overall signs score.

**Table 8.** Ocular signs related to number of administered eye drops per day among glaucoma patients.

Number of drops	Number of patients (%)	Eyelid redness	Conjunctival redness (SILK scale)	Corneal fluorescein staining (Oxford scale)	Conjunctival fluorescein staining (Oxford scale, combined nasal & temporal)	fBUT (seconds)^a^	Schirmer's test (millimeters)	Overall signs score
1–2	377 (66.9)	**0.9**	**1.9**	1.2 *	2.5	5.7	12.4	**10.4**
3–4	70 (12.4)	**0.7**	**1.8**	1.2	3.1 **	6.2	11.4	**10.6**
5–6	100 (17.7)	**1.2**	**2.0**	**1.5**	**3.2**	4.9	12.6	**12.0**
7–12	17 (3.0)	**1.6**	**2.8**	**2.5**	4.1 **	**2.4**	11.4	**16.4**
Total	564							
0 (controls)	51	0.04	1.2	0.8	2.0	7.3^b^	11.9	7.4

^a^ *n* = 557.

^b^ *n* = 48.

*Denotes statistical significance (Mann–Whitney) compared to controls with *p* < 0.05.

**Denotes statistical significance compared to controls with *p* < 0.01.

Bolded denotes statistical significance compared to controls with *p* < 0.001.

**Table 9.** Ocular symptoms related to number of administered eye drops per day among glaucoma patients.

Number of drops	Number of patients (%)	Irritation/burning/stinging	Itching	Foreign body sensation	Tearing	Dry eye sensation	Symptom sum
1–2	375 (67)	0.64	0.64	0.62	0.30	0.88	3.08
3–4	70 (12)	0.77	0.61	0.49	0.54	0.94	3.36 *
5–6	100 (18)	0.78	0.56	0.70	0.49	1.10 **	3.63 *
7–12	17 (3)	0.82	0.53	1.29 *	0.53	1.47 **	4.65 *
Total	562						
0 (controls)	51	0.57	0.39	0.47	0.35	0.59	2.37

*Denotes statistical significance (Mann–Whitney) compared to controls with *p* < 0.05.

**Denotes statistical significance compared to controls with *p* < 0.01.

## Discussion

Most of the glaucoma patients in this study suffered from the signs and symptoms of OSD. This was most significant in those who were medicated with three active compounds (beta-blocker and prostaglandin fixed-dose combination plus carbonic anhydrase inhibitor) or combinations with brimonidine. The signs and symptoms of OSD correlated with the increasing number of active compounds and/or eye drops of the used medication. These findings are consistent with previous studies that have showed association between glaucoma medication and OSD.^8,21–22^ Interestingly, duration of topical glaucoma medication did not correlate with signs or symptoms of OSD, even though a long-term exposure to glaucoma eye drops has been associated with changes in the ocular surface, conjunctiva, and trabecular meshwork.^23–25^

During the recent years, special attention has been paid on the role of preservatives in glaucoma eye drops. BAC is the most used preservative in ocular drugs. It is effectively penetrating to the eye tissues and has long-term effects on them.^16^ In addition to its effects on the ocular surface that causes ocular signs and symptoms, the use of BAC-containing drugs has been directly linked to the failure of glaucoma surgery.^17–19^ Overall, preserved topical glaucoma medication has been shown to cause OSD signs and symptoms, whereas preservative-free medications have showed significantly less ocular toxicity.^7,9,13,23,26^ In the presence study, we found similar relation to preservative-free compounds. A recent glaucoma guideline recommends the use of preservative-free medication in patients suffering from OSD.^27^

According to glaucoma guidelines, monotherapy is the preferred type of medication in glaucoma care. In our study, 43% of all glaucoma patients were using only one active compound. More than half of the glaucoma patients were using combined medications with multiple active compounds and daily eye drops. Furthermore, European Glaucoma Society Guidelines recommend the maximum use of three active compounds and three–four eye drops per day.^27^ In our study, 2% of the glaucoma patients had more than three active compounds in their medication and 21% used more than four eye drops per day. Similar reports have been shown previously.^28–29^ In this respect, it is important to recognize that the increasing number of active compounds and administered eye drops per day is associated with increasing severity of OSD signs, as shown in this study.

The strengths of this study include the robust, prospective nature of it. All data were obtained collectively from private clinics outpatients. Study subjects that were willing to participate in the study were enrolled consecutively at each center avoiding selection bias. As the study was multicenter-based with a large number of patients, the study represents well private clinics, where a significant proportion of glaucoma patients are treated by private ophthalmologists in Finland. To our knowledge, this is the first study to do so with standardized methods that include reference images for the OSD signs and a single, independent study nurse with a fixed protocol regarding the OSD symptom questionnaire.

Our study has also some limitations. Although this study was prospective, it was blinded only for recording the OSD symptoms by a single, independent study nurse. The study was cross-sectional and assessed only patients attending private ophthalmologist clinics in Finland. Therefore, even though these results can represent outpatient patient in general, they cannot be directly compared to those obtained from public eye hospitals. The number of controls patients was rather low, although it is comparable to other subgroups when different types of medications and number of them and their installations were compared. We also included comparisons to reference groups with the least impact on the OSD signs and symptoms in supplementary material.

Our results demonstrate that multi-therapy is common among glaucoma outpatients. Also, the prevalence of OSD signs and symptoms among these patients is related to the number of active compounds and administered eye drops per day. There are significant differences between glaucoma medications. Beta-blockers and preservative-free prostaglandin show the least effect on both ocular signs and symptoms. Therefore, BAC likely contributes to the ocular surface problems. These findings suggest that the use of preservative-free glaucoma medication is highly recommended. Special attention should be paid on patients having OSD signs and symptoms on what type of medicine is prescript, and in case of multi-therapy, to select laser or surgical treatment instead of increasing topical medication.

## Supplemental Material

sj-tif-1-ejo-10.1177_11206721221144339 - Supplemental material for Ocular surface disease signs and symptoms of glaucoma patients and their relation to glaucoma medication in Finland Click here for additional data file. Supplemental material, sj-tif-1-ejo-10.1177_11206721221144339 for Ocular surface disease signs and symptoms of glaucoma patients and their relation to glaucoma medication in Finland by Minna Parkkari, Petri Purola and Hannu Uusitalo in European Journal of Ophthalmology

sj-tif-2-ejo-10.1177_11206721221144339 - Supplemental material for Ocular surface disease signs and symptoms of glaucoma patients and their relation to glaucoma medication in Finland Click here for additional data file. Supplemental material, sj-tif-2-ejo-10.1177_11206721221144339 for Ocular surface disease signs and symptoms of glaucoma patients and their relation to glaucoma medication in Finland by Minna Parkkari, Petri Purola and Hannu Uusitalo in European Journal of Ophthalmology

sj-docx-3-ejo-10.1177_11206721221144339 - Supplemental material for Ocular surface disease signs and symptoms of glaucoma patients and their relation to glaucoma medication in Finland Click here for additional data file. Supplemental material, sj-docx-3-ejo-10.1177_11206721221144339 for Ocular surface disease signs and symptoms of glaucoma patients and their relation to glaucoma medication in Finland by Minna Parkkari, Petri Purola and Hannu Uusitalo in European Journal of Ophthalmology

sj-docx-4-ejo-10.1177_11206721221144339 - Supplemental material for Ocular surface disease signs and symptoms of glaucoma patients and their relation to glaucoma medication in Finland Click here for additional data file. Supplemental material, sj-docx-4-ejo-10.1177_11206721221144339 for Ocular surface disease signs and symptoms of glaucoma patients and their relation to glaucoma medication in Finland by Minna Parkkari, Petri Purola and Hannu Uusitalo in European Journal of Ophthalmology

sj-docx-5-ejo-10.1177_11206721221144339 - Supplemental material for Ocular surface disease signs and symptoms of glaucoma patients and their relation to glaucoma medication in Finland Click here for additional data file. Supplemental material, sj-docx-5-ejo-10.1177_11206721221144339 for Ocular surface disease signs and symptoms of glaucoma patients and their relation to glaucoma medication in Finland by Minna Parkkari, Petri Purola and Hannu Uusitalo in European Journal of Ophthalmology

sj-docx-6-ejo-10.1177_11206721221144339 - Supplemental material for Ocular surface disease signs and symptoms of glaucoma patients and their relation to glaucoma medication in Finland Click here for additional data file. Supplemental material, sj-docx-6-ejo-10.1177_11206721221144339 for Ocular surface disease signs and symptoms of glaucoma patients and their relation to glaucoma medication in Finland by Minna Parkkari, Petri Purola and Hannu Uusitalo in European Journal of Ophthalmology
