# Supplementary figures and images for: Ocular surface disease signs and symptoms of glaucoma patients and their relation to glaucoma medication in Finland
Source: Eur J Ophthalmol. 2022 Dec 13;33(2):993–1002. doi: 10.1177/11206721221144339 (PMC9999283; doi:10.1177/11206721221144339)

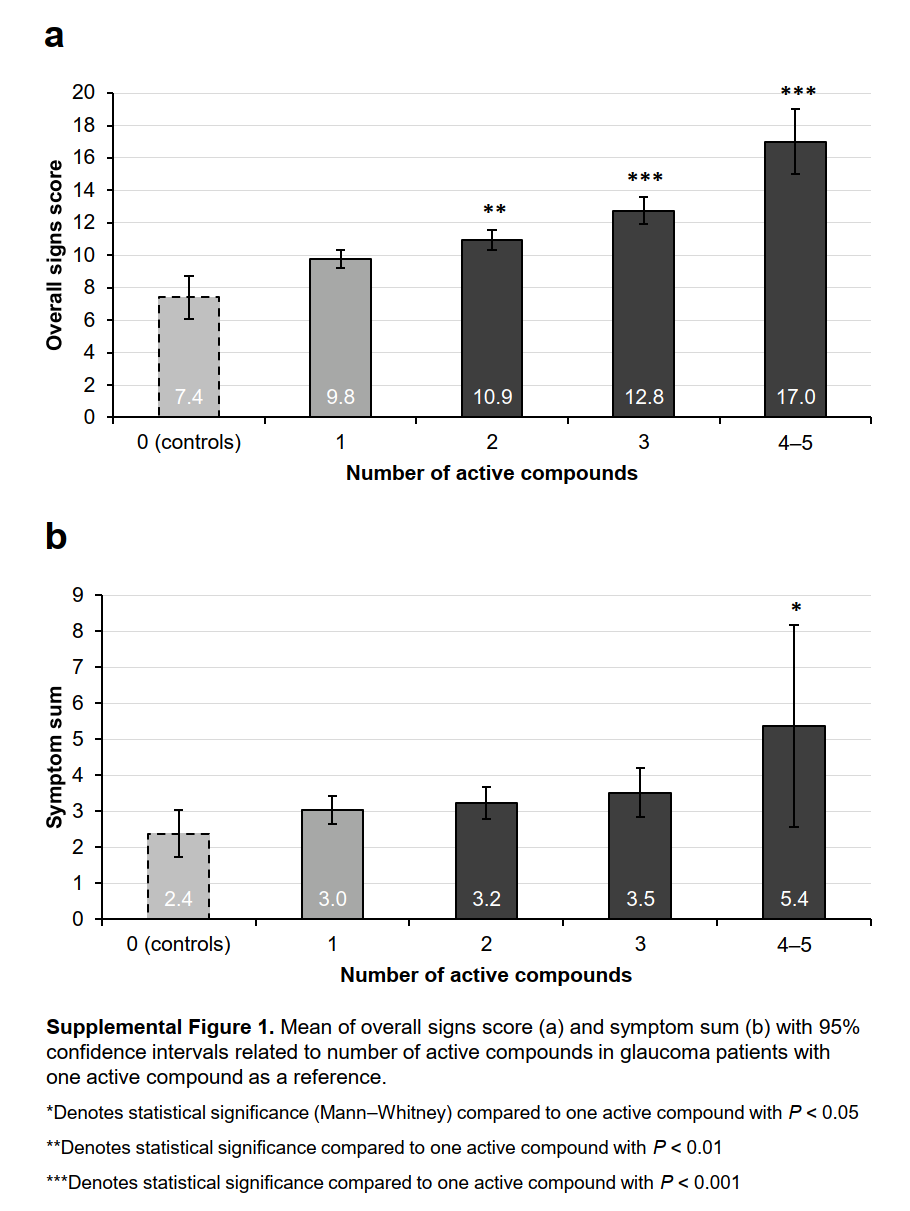

Supplement: sj-tif-1-ejo-10.1177_11206721221144339 - Supplemental material for Ocular surface disease signs and symptoms of glaucoma patients and their relation to glaucoma medication in Finland [file sj-tif-1-ejo-10.1177_11206721221144339.tif]

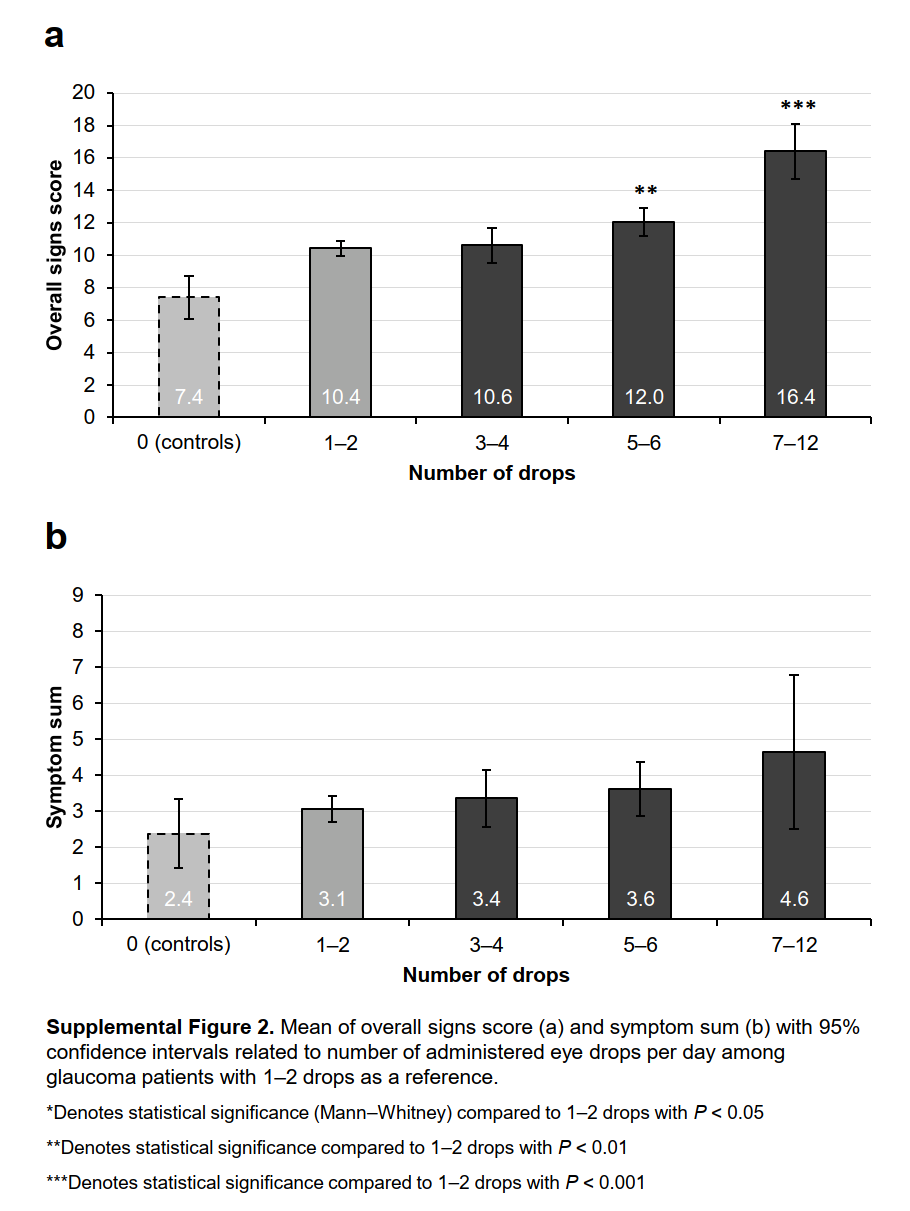

Supplement: sj-tif-2-ejo-10.1177_11206721221144339 - Supplemental material for Ocular surface disease signs and symptoms of glaucoma patients and their relation to glaucoma medication in Finland [file sj-tif-2-ejo-10.1177_11206721221144339.tif]
