# Supplementary material for: Ocular surface disease signs and symptoms of glaucoma patients and their relation to glaucoma medication in Finland
Source: Eur J Ophthalmol. 2022 Dec 13;33(2):993–1002. doi: 10.1177/11206721221144339 (PMC9999283; doi:10.1177/11206721221144339)
Supplement: sj-docx-3-ejo-10.1177_11206721221144339 - Supplemental material for Ocular surface disease signs and symptoms of glaucoma patients and their relation to glaucoma medication in Finland [file sj-docx-3-ejo-10.1177_11206721221144339.docx]

**Supplemental Table 1.** Prevalence of co-morbidities between glaucoma patients and controls.

|  | Patients (%) | Controls (%) | Chi-square p | Fisher’s test p |
| --- | --- | --- | --- | --- |
| Thyroid disease | 9.6 | 7.8 | 0.983 | 1.000 |
| Facial nerve paresis | 0.2 | 0.0 | 0.993 | 1.000 |
| Rosacea | 0.4 | 0.0 | 0.981 | 1.000 |
| Acne | 0.0 | 0.0 |  |  |
| Arterial hypertension | 41.7 | 33.3 | 0.719 | 0.298 |
| Coronary disease | 8.5 | 3.9 | 0.725 | 0.418 |
| Arrhythmia | 10.1 | 7.8 | 0.966 | 0.807 |
| Cardiac insufficiency | 3.9 | 0.0 | 0.559 | 0.244 |
| Diabetes mellitus | 10.5 | 15.7 | 0.725 | 0.243 |
| Osteoporosis | 3.2 | 3.9 | 0.994 | 0.678 |
| Rheumatoid arthritis | 2.3 | 2.0 | 0.999 | 1.000 |
| Sjögren's syndrome | 0.4 | 2.0 | 0.478 | 0.229 |
| Stevens-Johnson | 0.0 | 0.0 |  |  |
| Lyell syndrome | 0.0 | 0.0 |  |  |
| Other connective tissue syndrome | 1.2 | 0.0 | 0.887 | 1.000 |
| Fibromyalgia | 1.6 | 0.0 | 0.843 | 1.000 |
| Parkinson's disease | 0.4 | 0.0 | 0.981 | 1.000 |
| Psychiatric disease | 1.2 | 2.0 | 0.979 | 0.502 |
| Hormone replacement therapy | 5.7 | 3.9 | 0.965 | 1.000 |
| Cancer | 3.7 | 3.9 | 1.000 | 1.000 |
| Other relevant conditions; Work with display screen | 8.0 | 15.7 | 0.317 | 0.069 |
| Other relevant conditions; Use of contact lenses | 1.2 | 2.0 | 0.979 | 0.502 |
