# Supplementary material for: Ocular surface disease signs and symptoms of glaucoma patients and their relation to glaucoma medication in Finland
Source: Eur J Ophthalmol. 2022 Dec 13;33(2):993–1002. doi: 10.1177/11206721221144339 (PMC9999283; doi:10.1177/11206721221144339)
Supplement: sj-docx-4-ejo-10.1177_11206721221144339 - Supplemental material for Ocular surface disease signs and symptoms of glaucoma patients and their relation to glaucoma medication in Finland [file sj-docx-4-ejo-10.1177_11206721221144339.docx]

**Supplemental Table 2a.** Ocular signs related to glaucoma medication

| Medication | Number of patients (%) | Eyelid redness | Conjunctival redness (SILK scale) | Corneal fluorescein staining (Oxford scale) | Conjunctival fluorescein staining (Oxford scale, combined nasal & temporal) | fBUT (seconds)^a^ | Schirmer's test (millimeters) | Overall signs score |
| --- | --- | --- | --- | --- | --- | --- | --- | --- |
| Beta-blocker | 34 (6) | 0.2 | 1.4 | 1.1 | 2.7 | 6.6 | 12.0 | 8.8 |
| Prostaglandin, all | 204 (36) | **0.8** | 1.8 ** | 0.9 | 2.5 | 6.3 | 12.4 | 9.9 |
| Preserved prostaglandin | 160 (28) | **0.8** | **1.9** | 1.0 | 2.4 | 5.9 | 12.1 | 10.1 |
| Preservative-free prostaglandin | 44 (8) | **0.8** | 1.6 | 0.8 | 2.8 | 7.5 | 13.4 | 9.4 |
| Beta-blocker and prostaglandin ﬁxed-dose combination | 154 (27) | **1.0** | **2.0** | 1.5 | 2.6 | 4.9 * | 12.5 | 11.2 ** |
| Beta-blocker and other (than prostaglandin) ﬁxed-dose combination plus prostaglandin | 58 (10) | **1.3** | **2.1** | 1.2 | 3.4 | 5.3 | 12.1 | 12.0 ** |
| Beta-blocker and prostaglandin ﬁxed-dose combination plus carbonic anhydrase inhibitor | 37 (7) | **1.1** | **2.0** | **2.3** | 3.4 | **3.2** | 14.0 | **13.4** |
| Beta-blocker and other (than prostaglandin) ﬁxed-dose combination | 25 (4) | 0.4 | 1.6 | 1.4 | 3.3 | 6.5 | 10.4 | 10.4 |
| Prostaglandin plus carbonic anhydrase inhibitor | 16 (3) | **1.2** | 2.2 * | 1.6 | 2.5 | 5.4 * | 14.7 | 11.7 * |
| Beta-blocker and prostaglandin | 14 (4) | 0.7 ** | 1.6 | 0.6 | 2.6 | 8.3 | 9.0 | 9.3 |
| Combinations with brimonidine | 22 (3) | **1.4** | **2.8** | **2.3** | 3.7 | **2.9** | 11.9 | **15.1** |
| Total | 564 |  |  |  |  |  |  |  |

^a^*n* = 557

*Denotes statistical significance (Mann–Whitney) compared to beta-blocker with *P* < 0.05

**Denotes statistical significance compared to beta-blocker with *P* < 0.01

Bolded denotes statistical significance compared to beta-blocker with *P* < 0.001

**Supplemental Table 2b.** Ocular symptoms related glaucoma medication

| Medication | Number of patients (%) | Irritation/burning/stinging | Itching | Foreign body sensation | Tearing | Dry eye sensation | Symptom sum |
| --- | --- | --- | --- | --- | --- | --- | --- |
| Beta-blocker | 34 (6) | 0.59 | 0.26 | 0.59 | 0.59 | 0.85 | 2.85 |
| Prostaglandin, all | 202 (36) | 0.64 | 0.67 * | 0.60 | 0.27 * | 0.88 | 3.05 |
| Preserved prostaglandin | 158 (28) | 0.65 | 0.71 * | 0.68 | 0.30 | 0.89 | 3.23 |
| Preservative-free prostaglandin | 44 (8) | 0.59 | 0.52 | 0.32 | 0.14 * | 0.86 | 2.43 |
| Beta-blocker and prostaglandin ﬁxed-dose combination | 154 (27) | 0.68 | 0.69 * | 0.68 | 0.28 * | 0.90 | 3.23 |
| Beta-blocker and other (than prostaglandin) ﬁxed-dose combination plus prostaglandin | 58 (10) | 0.76 | 0.48 | 0.62 | 0.43 | 1.12 | 3.41 |
| Beta-blocker and prostaglandin ﬁxed-dose combination plus carbonic anhydrase inhibitor | 37 (7) | 0.84 | 0.78 ** | 0.70 | 0.62 | 0.78 | 3.73 |
| Beta-blocker and other (than prostaglandin) ﬁxed-dose combination | 25 (4) | 0.44 | 0.44 | 0.28 | 0.72 | 0.80 | 2.68 |
| Prostaglandin plus carbonic anhydrase inhibitor | 16 (3) | 1.20 | 0.53 | 0.40 | 0.40 | 1.00 | 3.53 |
| Beta-blocker and prostaglandin | 14 (4) | 0.43 | 0.50 | 0.43 | 0.36 | 1.36 | 3.07 |
| Combinations with brimonidine | 22 (3) | 0.96 | 0.73 | 1.46 * | 0.64 | 1.55 * | 5.32 * |
| Total | 562 |  |  |  |  |  |  |

*Denotes statistical significance (Mann–Whitney) compared to beta-blocker with *P* < 0.05

**Denotes statistical significance compared to beta-blocker with *P* < 0.01
