# Supplementary material for: Ocular surface disease signs and symptoms of glaucoma patients and their relation to glaucoma medication in Finland
Source: Eur J Ophthalmol. 2022 Dec 13;33(2):993–1002. doi: 10.1177/11206721221144339 (PMC9999283; doi:10.1177/11206721221144339)
Supplement: sj-docx-5-ejo-10.1177_11206721221144339 - Supplemental material for Ocular surface disease signs and symptoms of glaucoma patients and their relation to glaucoma medication in Finland [file sj-docx-5-ejo-10.1177_11206721221144339.docx]

**Supplemental Table 3a.** Ocular signs related to number of active compounds in glaucoma medication

| Number of active compounds | Number of patients (%) | Eyelid redness | Conjunctival redness (SILK scale) | Corneal fluorescein staining (Oxford scale) | Conjunctival fluorescein staining (Oxford scale, combined nasal & temporal) | fBUT (seconds)^a^ | Schirmer's test (millimeters) | Overall signs score |
| --- | --- | --- | --- | --- | --- | --- | --- | --- |
| 1 | 241 (43) | 0.7 | 1.8 | 0.95 | 2.5 | 6.3 | 12.3 | 9.8 |
| 2 | 207 (37) | 0.9 ** | 1.9 ** | **1.3** | 2.7 | 5.3 | 12.2 | 10.9 ** |
| 3 | 102 (18) | **1.2** | **2.1** | **1.7** | 3.4 ** | **4.3** | 12.6 | **12.8** |
| 4–5 | 14 (2) | **1.6** | **3.1** | **2.8** | **4.6** | **2.6** | 12.6 | **17.0** |
| Total | 564 |  |  |  |  |  |  |  |

^a^*n* = 557

**Denotes statistical significance compared to one active compound with *P* < 0.01

Bolded denotes statistical significance compared to one active compound with *P* < 0.001

**Supplemental Table 3b.** Ocular symptoms related to number of active compounds in glaucoma medication

| Number of active compounds | Number of patients (%) | Irritation/burning/stinging | Itching | Foreign body sensation | Tearing | Dry eye sensation | Symptom sum |
| --- | --- | --- | --- | --- | --- | --- | --- |
| 1 | 239 (43) | 0.63 | 0.61 | 0.60 | 0.32 | 0.88 | 3.04 |
| 2 | 207 (37) | 0.69 | 0.65 | 0.60 | 0.36 | 0.94 | 3.23 |
| 3 | 102 (18) | 0.75 | 0.59 | 0.67 | 0.52 | 1.00 | 3.52 |
| 4–5 | 14 (2) | 1.14 | 0.57 | 1.64 ** | 0.36 | 1.64 * | 5.36 * |
| Total | 562 |  |  |  |  |  |  |

*Denotes statistical significance (Mann–Whitney) compared to one active compound with *P* < 0.05

**Denotes statistical significance compared to one active compound with *P* < 0.01
