# Supplementary material for: Ocular surface disease signs and symptoms of glaucoma patients and their relation to glaucoma medication in Finland
Source: Eur J Ophthalmol. 2022 Dec 13;33(2):993–1002. doi: 10.1177/11206721221144339 (PMC9999283; doi:10.1177/11206721221144339)
Supplement: sj-docx-6-ejo-10.1177_11206721221144339 - Supplemental material for Ocular surface disease signs and symptoms of glaucoma patients and their relation to glaucoma medication in Finland [file sj-docx-6-ejo-10.1177_11206721221144339.docx]

**Supplemental Table 4a.** Ocular signs related to number of administered eye drops per day among glaucoma patients

| Number of drops | Number of patients (%) | Eyelid redness | Conjunctival redness (SILK scale) | Corneal fluorescein staining (Oxford scale) | Conjunctival fluorescein staining (Oxford scale, combined nasal & temporal) | fBUT (seconds)^a^ | Schirmer's test (millimeters) | Overall signs score |
| --- | --- | --- | --- | --- | --- | --- | --- | --- |
| 1–2 | 377 (67) | 0.9 | 1.9 | 1.2 | 2.5 | 5.7 | 12.4 | 10.4 |
| 3–4 | 70 (12) | 0.7 | 1.8 | 1.2 | 3.1 | 6.2 | 11.4 | 10.6 |
| 5–6 | 100 (18) | **1.2** | 2.0 * | 1.5 ** | 3.2 ** | 4.9 | 12.6 | 12.0 ** |
| 7–12 | 17 (3) | 1.6 ** | **2.8** | **2.5** | 4.1 ** | **2.4** | 11.4 | **16.4** |
| Total | 564 |  |  |  |  |  |  |  |

^a^*n* = 557

*Denotes statistical significance (Mann–Whitney) compared to 1–2 drops with *P* < 0.05

**Denotes statistical significance compared to 1–2 drops with *P* < 0.01

Bolded denotes statistical significance compared to 1–2 drops with *P* < 0.001

**Supplemental Table 4b.** Ocular symptoms related to number of administered eye drops per day among glaucoma patients

| Number of drops | Number of patients (%) | Irritation/burning/stinging | Itching | Foreign body sensation | Tearing | Dry eye sensation | Symptom sum |
| --- | --- | --- | --- | --- | --- | --- | --- |
| 1–2 | 375 (67) | 0.64 | 0.64 | 0.62 | 0.30 | 0.88 | 3.08 |
| 3–4 | 70 (12) | 0.77 | 0.61 | 0.49 | 0.54 * | 0.94 | 3.36 |
| 5–6 | 100 (18) | 0.78 | 0.56 | 0.70 | 0.49 | 1.10 | 3.63 |
| 7–12 | 17 (3) | 0.82 | 0.53 | 1.29 * | 0.53 | 1.47 | 4.65 |
| Total | 562 |  |  |  |  |  |  |

*Denotes statistical significance (Mann–Whitney) compared to 1–2 drops with *P* < 0.05
